# Effects of guidelines on adeno-tonsillar surgery on the clinical behaviour of otorhinolaryngologists in Italy

**DOI:** 10.1186/1472-6815-13-1

**Published:** 2013-01-07

**Authors:** Giovanni Motta, Sergio Motta, Pasquale Cassano, Salvatore Conticello, Massimo Ferretti, Bruno Galletti, Aldo Garozzo, Gennaro Larotonda, Nicola Mansi, Emilio Mevio, Gaetano Motta, Giuseppe Quaremba, Agostino Serra, Vincenzo Tarantino, Paolo Tavormina, Claudio Vicini, Maurizio Giovanni Vigili, Domenico Testa

**Affiliations:** 1 Institute of Otorhinolaryngology, University “Federico II”, Naples, Italy; 2 Institute of Otorhinolaryngology, Department of Preventive Medical Sciences, University “Federico II”, Naples, Italy; 3 Institute of Otorhinolaryngology, University of Foggia, Foggia, Italy; 4 Institute of Otorhinolaryngology, University of Torino, Torino, Italy; 5 Unit of Pediatric Otorhinolaryngology P.O.SS. Annunziata, Naples, Italy; 6 Institute of Otorhinolaryngology, University of Messina, Messina, Italy; 7 Institute of Otorhinolaryngology, University “Magna Graecia”, Catanzaro, Italy; 8 Unit of Otorhinolaryngology, Hospital “Madonna delle Grazie”, Matera, Italy; 9 S.C. of Otorhinolaryngology, Azienda Ospedaliera di Rilievo Nazionale Santobono-Pausillipon, Naples, Italy; 10 Unit of Otorhinolaryngology, Hospital “Fornaroli”, Magenta (Mi), Italy; 11 Institute of Otorhinolaryngology, II University of Naples, Naples, Italy; 12 Department of Forensic Medicine, University “Federico II”, Naples, Italy; 13 Institute of Otorhinolaryngology, University of Catania, Catania, Italy; 14 S.C. of Otorhinolaryngology “Giannina Gaslini”, Genoa, Italy; 15 Unit of Otorhinolaryngology, Pediatric Hospital “Regina Margherita S. Anna”, Torino, Italy; 16 Unit of Otorhinolaryngology, A.U.S.L. of Forlì, Forlì, Italy; 17 Unit of Otorhinolaryngology, Hospital “San Carlo - IDI”, Rome, Italy

**Keywords:** Tonsillectomy, Adeno-tonsillectomy, Otitis media, Guidelines

## Abstract

**Background:**

Several guidelines on adeno-tonsillar disease have been proposed in recent years and some discrepancies in relation both to clinical manifestations and indications for surgical treatment have emerged. The aim of the study was to verify what influence (adeno)-tonsillectomy guidelines have had on the clinical behaviour of ENT specialists in Italy. Our study is a retrospective and multi-centre case series with chart review.

**Methods:**

The survey involved 14,770 children, aged between the ages of 2 and 11, who had undergone adeno-tonsillar surgery between 2002 and 2008 in fourteen Italian tertiary and secondary referral centres. Anova test was used for the statistical analysis, assuming p < 0.05 as the minimum statistical significance value.

**Results:**

The frequency of adeno-tonsillar surgeries did not change significantly (p>0.05) during the study period and following the Italian policy document publication. Overall, adeno-tonsillectomy was the most frequent intervention (64.1%), followed by adenoidectomy (31.1%) and tonsillectomy (4.8%). The indications for surgery did not change significantly for each of the operations (p>0.05), with the exception of adeno-tonsillectomy in case of feverish episodes due to acute recurrent tonsillitis ≥ 5 without nasal obstruction (decreased p= 0.010) , even when the feverish episodes due to acute recurrent tonsillitis were < 5 over the last year. Nasal obstruction was associated with feverish episodes due to acute recurrent tonsillitis in 65.2% of operated cases, while otitis media had been diagnosed in 43.3% of the patients studied.

**Conclusions:**

The recommendations first developed in Italy in a 2003 policy document and then resumed in guidelines in 2008, were not implemented by ENT units involved in the survey. The study highlights the fact that the indications for adeno-tonsillar operations are based on the overall clinical presentation (comorbidity) rather than on a single symptom. Guidelines are necessary to give coherent recommendations based on both the findings obtained through randomized controlled trials and the data collected from observational studies.

## Background

Several guidelines (GL) on adeno-tonsillar disease and its treatment have been proposed in recent years by scientific associations [1,2] or by institutions specifically delegated to the drafting of such documents [3,4] in order to standardize and rationalize indications for (adeno)-tonsillectomy in children.

In Italy too, this topic has been the subject of a careful study, carried out by a committee of experts appointed by the Ministry of Health. In April 2003 the Italian National System for Guidelines published a Policy Document (PD) on adeno-tonsillar disease and its related surgical treatments [5] and in March 2008, this document was revised and resubmitted as GL [6]. Briefly the principal indications developed in both documents are five or more feverish episodes due to acute recurrent tonsillitis (FEART) in a single year and the presence of obstructive sleep apnoea syndrome (OSAS).

Moreover, some of the currently known GL on adeno-tonsillar disease have been revised over the years with significant modifications to both the key parameters, related to surgical interventions, and the forcefulness of recommendations [1-4].

The apparent discrepancies in the different GL relate mainly to those clinical manifestations which give an indication for surgery, but also to the number and strength of the various recommendations contained in these documents. The true value of the criticism raised against the GL should be evaluated, keeping in mind that verification was also sought by the American Academy of Otolaryngology-Head and Neck Surgery (AAOHNS), that acknowledged the requirement for research to assess “the impact and use of the guidelines by determining how the guidelines translate to performance measurements and performance improvement” [2].

The degree of acceptance of the recommendations in the surgical guidelines set out by Italian ENT specialists in the mentioned documents is not known to date.

The present study intends to determine whether, as a result of the recommendations concerning the clinical indications to (adeno)-tonsillectomy contained in the 2003 PD [5] and confirmed by the 2008 GL [6], the frequency of adeno-tonsillar surgery has changed over the last years in the ENT units included in the survey and if the otolaryngologists involved have modified their behaviour in line with the indications for these surgical interventions.

## Methods

Fourteen Italian tertiary and secondary referral centers including ENT units were recruited for the present research, 10 of which (6 university hospitals and 4 hospitals) operating in general hospitals and 4 in paediatric hospitals.

The participating centres were selected from a previous epidemiologic survey [7] conducted by means of a questionnaire on number and type of adeno-tonsillar surgical intervention which had been sent to ENT paediatric surgical centres in Italy. 14 centres, distributed on the Italian territory, answered fully. Our observation refers to data obtained by the recruited centres and thus represent the clinical behaviour of the ENT specialists involved in the survey. None of the researchers of this survey was involved in the authorship of the guidelines.

The present research was performed in accordance with the institutional review board guidelines, as well as the Helsinki Declaration of 1983, and it has been reviewed and approved by the Ethics Committee of the School of Medicine Federico II, Naples, Italy (n^o^ 42/12).

The medical directors of the ENT units involved were first requested to fill in a questionnaire regarding the medical records of the years 2002, 2004, 2006, 2008 related to children, aged between 2 and 11, who had undergone adeno-tonsillar surgery (Adeno-Tonsillectomy, AT; Tonsillectomy, T; Adenoidectomy, A).

With regards to tonsillar surgery, the data provided by the recruited centres included only tonsillectomy - to the best of our knowledge - tonsillotomy is rarely performed in Italy.

After which, they were asked to identify the indications provided for each of the three operations studied, on the basis of five different clinical presentations (Table 1) both for AT and T then for A, with reference to the number of FEART suffered over the last year (≥ 5, in accordance with Italian GL; or < 5, not in accordance with Italian GL) , the presence of nasal respiratory obstruction (NRO) in association with FEART (AT and T) or even as exclusive clinical manifestation (A), and elevated Antistreptolysin O (ASO) titer with distant disorders possibly due to group A β hemolytic streptococcus infection (GABHS).

**Table 1:** The indications to pharyngeal surgical interventions provided in the questionnaire

**AT and T**	**A**
1. < 5 FEART a year without NRO *	1. < 5 FEART a year without NRO
2. < 5 FEART a year with NRO *	2. < 5 FEART a year with NRO
3. ≥ 5 FEART a year without NRO °	3. ≥ 5 FEART a year without NRO
4. ≥ 5 FEART a year with NRO °	4. ≥5 FEART a year with NRO
5. GABHS infection °	5. NRO

*AT* = Adeno-Tonsillectomy; *T* = Tonsillectomy; *A*= Adenoidectomy; *FEART* = Feverish Episodes due to Acute Recurrent Tonsillitis; *NRO* = Nasal Respiratory Obstruction. *GABHS* = Group A β Hemolytic Streptococcus.

* not in accordance with Italian GL; ° in accordance with Italian GL.

As far as the number of FEART is concerned, the patients were divided into two groups depending on the number of feverish episodes (≥ 5 or < 5) suffered in the 12 months preceding the first medical examination. The possibility of GABHES infection was indicated if the ASO titer was double or more the upper limit of the normal range, regardless of test technique used.

The severity of NRO due to pharyngeal lymphatic tissue hyperplasia (frequent colds with occasional or persistent nasal discharge; open mouth respiration during daytime and/or during night sleep, with or without association of brief and sporadic episodes of sleep apnoea; snoring; prolonged and frequent episodes of sleep apnoea) was not evaluated as not useful to the aim of the present study. As for tympanic inflammations, the presence of persistent and/or recurrent effusive otitis media (EOM) had to be documented by clinical examination and by type B tympanogram recorded at least three times in the 12 months prior to surgical intervention; the diagnosis of acute recurrent otitis media (AROM) was formulated in the case of at least 4 acute episodes over the previous year, or 3 episodes occurring over the previous six months. Anova test was used for the statistical analysis (statistical package SPSS, 10.3 version; Boston – USA) assuming p < 0.05 as the minimum statistical significance value.

## Results

**Figure 1:**
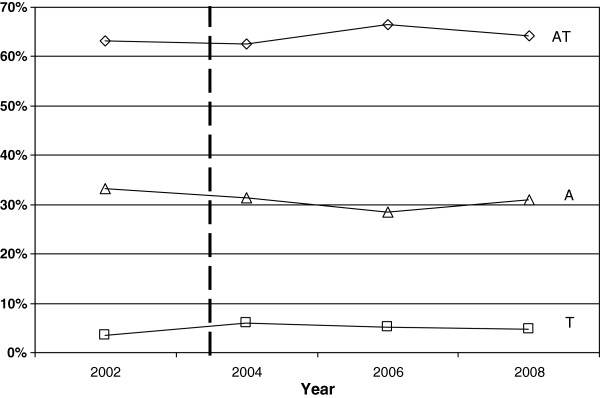
**Trends of operated cases.** The dashed line indicates the introduction of PD in 2003 in Italy. AT = Adeno-Tonsillectomy; T = Tonsillectomy; A= Adenoidectomy.

The fourteen units involved in the study provided data relative to 14,770 subjects operated on during the study period, of whom 9,469 cases underwent AT, 714 T and 4,587 A. Overall, AT represented the most frequent operation (64. 1%), followed by A (31.1%) and finally T (4.8%) with the lowest incidence (Figure 1). None of the three types of pharyngeal operations underwent any significant variation over the study years (p>0.05).

The indications for AT remained fairly constant in the study period (p> 0.05), except for FEART ≥5 in the previous year without NRO (decreased; p= 0.010) , even when FEART were < 5 over the previous year with ( p = 1) or without NRO (p = 0.539). In 76.1% of cases the indication for AT depended on the association between FEART with NRO (Table 2).

**Table 2:** Trend in absolute numbers and percentages of AT, T and A

	***Study year***					P*
	**2002**	**2004**	**2006**	**2008**	**Total 2002-2008**	
***AT***						
< 5 FEART without NRO	56 (2.3%)	47 (2.0%)	43 (1.8%)	44 (2.0%)	190 (2.0%)	0.539
< 5 FEART with NRO	855 (34.5%)	955 (41.3%)	914 (37.5%)	809 (36.1%)	3533 (37.3%)	1
≥ 5 FEART without NRO	344 (13.9%)	309 (13.4%)	268 (11.0%)	235 (10.5%)	1156 (12.2%)	0.010
≥ 5 FEART with NRO	990 (40.0%)	779 (33.7%)	984 (40.4%)	920 (41.0%)	3673 (38.8%)	0.637
GABHS infection	233 (9.4%)	223 (9.6%)	227 (9.3%)	234 (10.4%)	917 (9.7%)	0.553
***Total AT***	2478	2313	2436	2242	**9469**	0.307
	**(100%)**	(100%)	(100%)	(100%)	(100%)	
***T***						
< 5 FEART without NRO	6 (4.3%)	24 (10.9%)	9 (4.8%)	3 (1.8%)	42 (5.9%)	0.605
< 5 FEART with NRO	9 (6.5%)	25 (11.3%)	23 (12.4%)	20 (11.8%)	77 (10.8%)	0.132
≥ 5 FEART without NRO	72 (52.2%)	106 (48.0%)	74 (39.8%)	71 (42.0%)	323 (45.2%)	0.109
≥ 5 FEART with NRO	40 (29.0%)	46 (20.8%)	58 (31.2%)	65 (38.5%)	209 (29.3%)	0.317
GABHS infection	11 (8.0%)	20 (9.0%)	22 (11.8%)	10 (5.9%)	63 (8.8%)	0.845
***Total T***	138	221	186	169	**714**	0.784
	(100%)	(100%)	(100%)	(100%)	**(100%)**	
***Total AT+T***	2616 (100%)	2534	2622	2411	**10183**	0.309
	(100%)	(100%)	(100%)	(100%)	**(100%)**	
***A***						
< 5 FEART without NRO	285 (21.9%)	237 (20.4%)	293 (28.1%)	265 (24.6%)	1080 (23.5%)	0.373
< 5 FEART with NRO	379 (29.1%)	282 (24.3%)	254 (24.3%)	277 (25.7%)	1192 (26.0%)	0.508
≥ 5 FEART without NRO	25 (1.9%)	17 (1.5%)	9 (0.9%)	13 (1.2%)	64 (1.4%)	0.225
≥ 5 FEART with NRO	241 (18.5%)	259 (22.3%)	236 (22.6%)	211 (19.6%)	947 (20.6%)	0.592
NRO	373 (28.6%)	367 (31.6%)	252 (24.1%)	312 (28.9%)	1304 (28.4%)	0.689
***Total A***	1303	1162	1044	1078	**4587**	0.113
	(100%)	(100%)	(100%)	(100%)	**(100%)**	

*AT* = Adeno-Tonsillectomy; *T* = Tonsillectomy; *A*= Adenoidectomy; *FEART* = Feverish Episodes due to Acute Recurrent Tonsillitis; *NRO* = Nasal Respiratory Obstruction. *GABHS* = Group A β Hemolytic Streptococcus.

The indications for T were relatively irregular in relation to the extremely low number of cases (4.8% of the study patients). In this group the most frequent indication (74.5% on average) was constituted by FEART ≥5, of which 40.1% were subjects in whom surgical indication arose as the consequence of the simultaneous presence of FEART and NRO. The indication for surgical treatment was due to FEART <5 without NRO (Tab. 2), with a percentage varying during the study from 1.8% to 10.9% of these cases.

The indications for A predominantly involved patients with NRO (75.0%). The simultaneous presence of FEART and NRO brought 46.6% of these cases to surgery (Table 2).

Overall, out of 14,770 patients who underwent surgical treatment, 6396 (43.3%) complained of recurrent AROM and/or EOM; the frequency of otitis media in patients who underwent surgical interventions showed no significant variation (p>0.05) during the years of the study.

## Discussion

The principal aim of our survey was to verify the degree of acceptance of the Italian GL by specialists directly involved in the research. In this regard the authors of the Italian GL assumed that the dissemination of the PD was adequate and that a reduction in the number of tonsillar interventions and in the variability of tonsillectomy from area to area had occurred [6]. This assertion is, however, partially in contrast with the data of Fedeli et al. who found a wide heterogeneity in AT rates according to nationality and local health units in Veneto (Italy) after the publication of the PD in 2003 [8].

During the study period (from 2002, one year before the PD publication, to 2008, 5 years after the publication of the document) the overall number of (A)T and A effected in the recruited units showed no significant variation. This data tends to exclude the real impact of the recommendations formulated in the 2003 PD on the clinical behaviour of ENT specialists included in the survey, at least for the containment of adeno-tonsillar operations.

As previously reported, PD is widespread in Italy, and it can be assumed that most of the ENT specialists - and among them certainly the specialists involved in the present investigation - were well aware of the recommendations expressed in these documents.

The clinical manifestations that generally lead to indications for adeno-tonsillar surgery are very differently interpreted by the various GL (Table 3) [1-6]. As for the FEART, almost all GL refer to the study of Paradise et al [9], according to which (A)T should be proposed in cases of 7 or more episodes of tonsillitis over the previous year, 5 or more episodes per year in the previous 2 years, or even 3 or more episodes per year in the previous 3 years. Nevertheless, the indications reported in the cited GL [1-6] do not always adhere to these criteria: surgery has in fact been proposed after 5 or more FEART in the previous year in the GL published in 1999 by Scottish Intercollegiate Guidelines Network (SIGN) [3]; after 3 or more episodes in the Clinical Indicators Compendium published by AAOHNS in 2000 [1]; after 5 or more episodes in the PD (2003) [5] and GL (2008) [6] published in Italy. Only in 2010 SIGN [4] and in 2011 AAOHNS [2] proceeded to a new formulation of the GL adopting the criteria formulated by Paradise et al. in 1984 [9].

**Table 3:** Main indications for T and AT and number of recommendations related to to surgical management in GL on adeno-tonsillar disease

**GL**	**Number of FEART**	Strength of the recommendation*	**NRO**	**Otitis media**	**Number of recommendations**
**SIGN: 1999**^**3**^	≥ 5 a year	C/A-C	Not mentioned	Not mentioned	3
**AAOHNS: 2000**^**1**^	≥ 3 a year	-	Hypertrophy causing upper airway obstruction	RAOM or EOM	8
**PNLG: 2003**^**5**^	≥ 5 a year	A/A-D	OSAS	Surgery is not recommended	53
**SNLG 2008**^**6**^	≥ 5 a year	A/A-D	OSAS	Surgery is not recommended	79
**SIGN: 2010**^**4**^	≥ 7 in the last year	D/A-D	Not mentioned	Not mentioned	4
	≥ 5 in the last 2 years				
	≥ 3 in the last 3 years				
**AAOHNS: 2011**^**2**^	≥ 7 in the last year	B/A-D	Sleep Disordered Breathing (SDB), including snoring, mouth breathing, pauses in breathing, etc. (“*comorboid conditions”)*	Not mentioned	10
	≥ 5 in the last 2 years				
	≥ 3 in the last 3 years				

*GL* = Guidelines; *FEART* = Feverish Episodes due to Acute Recurrent Tonsillitis; *NRO* = Nasal Respiratory Obstruction. *SIGN* = Scottish Intercollegiate Guidelines Network; *AAOHNS* = American Academy of Otolaryngology-Head and Neck Surgery; *PNLG* =Programma Nazionale Linee Guida; *SNLG* = Sistema Nazionale Linee Guida. Strength of the recommendation related to the number of FEART.

In the present study, the indication for AT and T was given in 37.7% of the patients in relation to a number of FEART <5, with (35.4%) or without (2.3%) NRO. Furthermore, the incidence of patients who underwent T with FEART <5 without other clinical manifestations increased to 10.9% in 2004, suggesting that the ENT surgeons involved in the present survey did not apply the recommendations formulated in the PD [5].

Some GL do not consider NRO and its clinical relationship with sleep-disordered breathing, while others approach this issue in very different ways. For instance, the GL published by SIGN [3,4] does not mention this aspect at all, excluding it from its assessment. On the other hand, the AAOHNS [1] GL (2000) refers to all adeno-tonsillar hypertrophies as responsible for obstructive respiratory disease. Italian PD [5] and GL [6] prefer to give relevance only to cases with OSAS documented instrumentally, such as pulse oxymetry and polysomnography. Finally, the AAOHNS GL of 2011 [2] draws attention to children in whom sleep-disordered breathing, comorboid conditions (growth retardation, poor school performance, enuresis and behavioral problems) and tonsillar hypertrophy are susceptible to improvement after surgical treatment.

In the present study NRO was frequently observed in patients, often in association with FEART (65.2%). Clinical manifestations related to NRO as a whole should not be ignored by GL or similar documents, as they could be linked to several behavioural modifications that necessarily influence the QoL, creating anxiety in parents [10].

The different GL also show discrepancies regarding the role of adeno-tonsillar surgery in the management of otitis media: the GL of the SIGN [3,4] and that developed by the AAOHNS in 2011 [2] do not consider otitis media as an indication for surgery. The GL of the AAOHNS published in 2000 [1] recommend A associated with T for AROM and EOM. Otherwise, the Italian PD [5] and GL [6] consider the suitability of A only in cases when EOM is non responsive to medical treatments, associated with chronic adenoiditis. In our study, 43.3% of the patients who underwent surgical treatments had tympanic inflammations, suggesting a strong correlation between otitis media and adeno-tonsillar disease.

The indications for T, with or without A, derived in most of the study cases, from the concurrent presence of several clinical manifestations related to adeno-tonsillar disease (comorbidity). For instance, association between FE and NRO was observed in 73.6% of the patients who underwent AT and T: these data raise many doubts concerning the real importance of defining precise parameters for each of these clinical manifestations and show that, for surgical indication, each clinical manifestation alone is less conclusive than their association.

As is well known, the recommendations of the GL must adhere to the principles of Evidence-Based Medicine, which assume more strength and credibility when based on studies with a high level of evidence and, in particular, on randomized controlled studies.

The investigations of Paradise et al. [9], which were followed by subsequent studies by the same group [11] and by those of other researchers [12,13], were undoubtedly essential in this topic. However, several authors have raised objections to these studies [14-16], pointing out the possibility of bias in the investigation technique and the scarse acknowledgement of relations existing between the different pathological conditions related to adeno-tonsillar disease [17-20].

Arguing that surgical treatment is indicated only if FEART are>7 [9] is simplistic for us: such treatment could be considered unquestionably valid also in cases with a lower number of feverish episodes, but with signs of significant upper airway obstruction, recurrent tympanic inflammations or positivity for GABHES infection [21]; in this respect, the criticism of Darrow and Siemens [15], who analyzed the various symptoms of chronic adeno-tonsillitis, and the remarks of van den Akker et al. [22] on the possibilities of various therapies in order to treat the overall clinical picture, seem to us particularly relevant.

With regard to the analysis of the relations between GL and otitis media, we should refer to the study of Paradise et al. [11] 1999, who discussed the statistical assumptions of their study with particular precision. Unfortunately, the considerable dispersion of data reduces the reliability of the reported remarks for the present study too. Moreover, the fact that the majority of patients (72.6%) of the 3-way trial underwent a surgical treatment (A or AT), which is considered risky by the authors, without there having been any clear indication for surgery, and that 66.2% of patients of the 2-way trial were operated on for AT without any nasal obstruction attributable to large adenoid, raises some concerns.

These are biases that stand in contrast to each other and negatively effect the results and the conclusions of the investigation, especially with regards to children affected by clinical manifestations suggestive of adeno-tonsillar disease and with a more severe clinical course [23]. Some of the comments made in the study of Paradise et al., regarding the lack of representativeness of the series, the high incidence of cases lost to follow-up, the high proportion of control cases that underwent surgical treatment during the investigation, the inclusion of these patients in control cases, could be extended to other investigations [24]. Results obtained from this study regarding the scant acceptance of Italian GL recommendations by the ENT specialists involved in the survey, may show the reluctance on behalf of some specialists to change a rooted clinical behaviour. Nevertheless such data demonstrates that the variability of surgical indications suggested by ENT specialists is based on the presence of several contemporaneous clinical manifestations, a characteristic aspect of adeno-tonsillar disease which is not given sufficient attention in GL written in Italy or in other countries.

The results of our study naturally do not intend to formulate strict rules or even substitute the GL. They are intended to point out that the recommendations for tonsillectomy proposed by GL do not always coincide with the clinical indications, especially in the presence of comorbidity.

In our opinion, the limitations in the studies based on RCTs, imposed by the stringent rules governing patient recruitment and the assessment criteria, mean that results do not accurately reflect the clinical reality in some application areas, such as the present one.

Rosenfeld, co-editor with Bluestone of a meticulous monography on otitis media [25], notes that it is dangerous to classify all the published randomized trials as high quality and all the observational studies as poor quality research and that randomization can not substitute imprecise selection criteria, poorly defined objectives, inadequate follow-up, or reduced compliance to treatment. This last remark supports the view of Schon and Stanley [26] on the need to consider the RCTs and the studies based on clinical experience or observational studies as complementary.

## Conclusions

The present study analyses the effects that guidelines on (adeno)-tonsillar surgery had on the clinical behavior of ENT specialists in Italy. The results of this study show that the recommendations in these documents did not led to changes in practice for these operations, in the recruited ENT units. Current guidelines, such as the Italian GL, generally recommend (adeno)-tonsillectomy only if at least 5 feverish episodes have occurred in the last year. In most of the patients studied, however, the indication for surgery was the presence of comorbidity represented by various and concurrent clinical manifestations. Although some of the published guidelines mention comorbidity, none of them report recommendations with specific reference to this issue. Our findings raise many doubts on the real importance of defining precise parameters for each of the clinical manifestations related to adeno-tonsillar disease and on the true correspondence to clinical reality of the recommendations laid down in some of these documents, which might justify the apparent disregard for them in current practice. We consider it appropriate, therefore, to propose a revision of the guidelines to unify their orientations in order to guarantee patients’ health and to protect the prestige and professionalism of surgeons, too often involved in legal disputes that have only speculative purposes.

Further studies will certainly be needed to critically analyze GL on (adeno)-tonsillectomy (and related topics) and to determine the reasons that lead ENT practitioners to deviate from the recommendations contained in these documents.

## Abbreviations

GL: Guidelines; PD: Policy Document; ENT: Ear-Nose-Throat; A: Adenoidectomy; T: Tonsillectomy; AT: Adeno-Tonsillectomy; FEART: Feverish Episodes due to Acute Recurrent Tonsillitis; NRO: Nasal Respiratory Obstruction; GABHS: Group A β haemolytic streptococcus infection; EOM: Effusive Otitis Media; AROM: Acute Recurrent Otitis Media; SIGN: Scottish Intercollegiate Guidelines Network; AAOHNS: American Academy of Otolaryngology-Head and Neck Surgery; PNLG: Programma Nazionale Linee Guida; SNLG: Sistema Nazionale Linee Guida.

## Competing interests

The authors declare that they have no competing interests.

## Authors’ contributions

GM conceived the study, participated in its design, drafted the manuscript; SM made substantial contributions to conception and design; PC made substantial contributions to acquisition of data; SC made substantial contributions to acquisition of data; MF approved the final version for publication; BG made substantial contributions to acquisition of data; AG approved the final version for publication; GL made substantial contributions to acquisition of data; NM made substantial contributions to acquisition of data; EM made substantial contributions to acquisition of data; GM made substantial contributions to conception and design and interpretation of data; GQ made substantial contributions to analysis of data; AS made substantial contributions to acquisition of data; VT made substantial contributions to acquisition of data; PT made substantial contributions to acquisition of data; CV made substantial contributions to acquisition of data; MGV drafted the manuscript; DT made substantial contributions to design and interpretation of data. All authors read and approved the final manuscript.

## Pre-publication history

The pre-publication history for this paper can be accessed here:

http://www.biomedcentral.com/1472-6815/13/1/prepub
